# Correction

**DOI:** 10.1111/1759-7714.14810

**Published:** 2023-01-31

**Correction to Pleural homocysteine for malignant pleural effusion: A prospective and double‐blind diagnostic test accuracy study**

In Xi‐Shan Cao et al.^1^ the following errors were published on page 2357.

In the version of Figure 1 initially published, there is an error in the reason for excluding patients in the Hohhot cohort. It should be revised as: Excluded: *n* = 34; with unknown etiology: *n* = 11; consent was withdrawn: *n* = 2; diagnosis can be made with history: *n* = 4; insufficient specimen: *n* = 17. In addition, some MPE patients were diagnosed with evidence of primary or secondary cancer, and benign pleural effusion can be excluded by treatment response and follow‐up. The pleural fluid specimens were stored between −70 and −80°C.

In Table 1, the causes of MPE in the Hohhot cohort were: lung cancer (*n* = 47); mesothelioma (*n* = 4); gastric cancer (*n* = 2); breast cancer (*n* = 1); lymphoma (*n* = 1); synovial sarcoma (*n* = 1); unknown (*n* = 1). In the Changshu cohort, the causes of MPE were: lung cancer (*n* = 21); mesothelioma (*n* = 1); unknown (*n* = 4).

Figure 5 is incorrect and should be revised as the following figure.
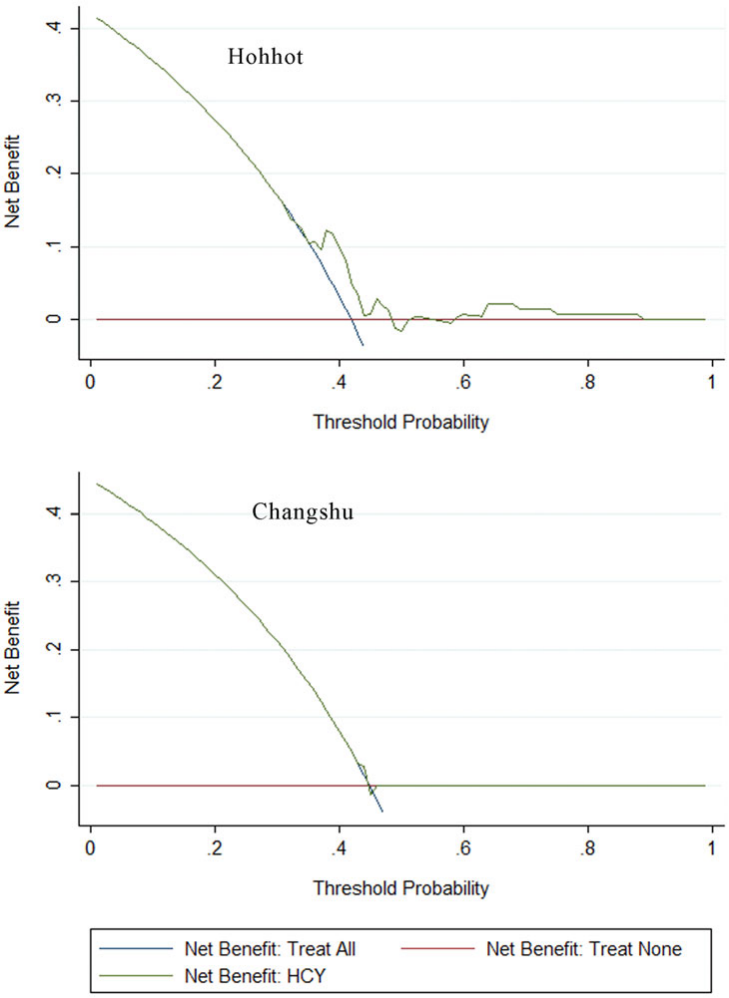


These changes do not affect the conclusion of the study.

The authors apologize for the errors and any inconvenience these may have caused.
